# Knowledge mapping of vocational education and training research (2004–2020): a visual analysis based on CiteSpace

**DOI:** 10.1038/s41598-023-49636-7

**Published:** 2023-12-15

**Authors:** Yumi Tian, Jiayun Liu, Xin Xu, Xueshi Wu

**Affiliations:** College of Education, Jiangxi Science and Technology Normal University, Nanchang, China

**Keywords:** Psychology, Energy science and technology

## Abstract

The study aims to analyze the leading researchers of vocational education and training from dimensions of individuals, institutions and countries. This article utilises the scientific information measurement software—CiteSpace—to conduct a scientometric analysis of 2,024 articles on vocational education and training from the Web of Science (W.o.S.). According to the research results, some useful conclusions can be drawn as follows: (1) vocational education and training research has become interdisciplinary and subject involved are “psychology”, “sociology”, “economics” and “pedagogy”; (2) the United States, the Netherlands and Australia make the majority of contributions and there are numerous collaborations among countries; (3) Univ Amsterdam, Univ Utrecht and Univ Melbourne were the main research institutions; (4) J Vocat Educ Train, Rev Educ Res, Thesis Elev, Econ Educ Rev and J Educ Work were the top 5 highly cited journals; (5) “Engagement”, “Program”, “Self-efficacy”, “High school”, “Predictor” and “Labor market” have become major research hotspots currently.

## Introduction

Nowadays the importance of vocational education and training has been highlighted by the rapid economic and social development with relatively mature vocational education and training systems established in countries like the United States, Germany, Australia, the United Kingdom and the Netherlands. Subsequently, researchers with academic backgrounds in economics^1–3^, management^4–6^, and information technology^7–9^ around the world have paid close attention to the courses^10,11^, professions^12^, entrepreneurship^13,14^, skills^15,16^ and evaluations^17^ of this field.

Social changes are manifested in greater mobility of workers, shifting labor markets, frequent changes of professions, the disappearance of several professions and the emergence of new ones^18,19^. The emergence of artificial intelligence tools, which are transforming the entire landscape of the labor market, is becoming a significant risk and challenge. The need to learn constantly and throughout life and the instability of professional development make vocational education and training the most important and defining sector of education^20,21^. Many researchers agree that its role will constantly grow and cover all sectors of professional implementation^22,23^. Bibliographic analysis of the field of research in the field of vocational education is rapidly evolving, but there are fewer review works on this sector than it requires^24^.

Vocational education and training are studied from the point of view of assistance from the state administration or municipalities in the retraining of employees^6,16^; many country case studies provide insight into the differences in the educational context of individual countries^2,3,11,14^. Experimental studies on combining vocational education and work or other types of employment make it possible to assess the potential of problems that require solutions^25–27^. The use of technical and digital tools within vocational education is also being devoted to more and more research^9,28,29^.

The existing research results have laid an important foundation for the reform and development of vocational education and training. However, vocational education and training still have a series of problems that need to be solved, such as the large gap between the skill supply of vocational education and the skill demand of the labor market, and the low enthusiasm of enterprises to participate in vocational education and training^4,9^. In addition, although many scholars use a variety of research methods from different dimensions to explore related issues of vocational education and training, few studies have investigated vocational education and training comprehensively and systematically^23,30^. There is an urgent need for bibliometric analysis to identify areas of development, areas of greatest interest among researchers, and stratification of research by country, institution, and area. This will allow the efforts of new researchers to be more targeted and their quality improved.

The objectives of this study are as follows:

1. Analyze the leading researchers of vocational education and training from dimensions of individuals, institutions and countries;
2. Figure out the distribution of journals related to vocational education and training;
3. Delve into the main research topics and knowledge structure in this field;
4. Aggregate the research hotspots and frontiers in this field.

## Methods

### Data

The data used in the study were obtained through advanced retrieval from the Web of Science Core Collection (WOSCC): "Science Citation Index Expanded (SCI-E) (2004–2020)"; Social Science Citation Index (SSCI) (2004–2020)"; "Conference Proceedings Citation Index-Science (CPCI-S) (2004–2020)". These data are intended to provide comprehensive, scientific and systematical research on the existing literature related to vocational education and training. The retrieval strategy was as follows: TS = ("Vocational Education" or "Technical and Vocational Education and Training" or "Technical Education" or "Technical and Further Education" or "Technical and Further Education" or ((VET) or (TVET) and (education))), and with language options of "English" and literature type selection "Article". Here VET is the "Vocational Education and Training" abbreviation and TVET "Technical and Vocational Education and Training" abbreviation.

**Figure 1:**
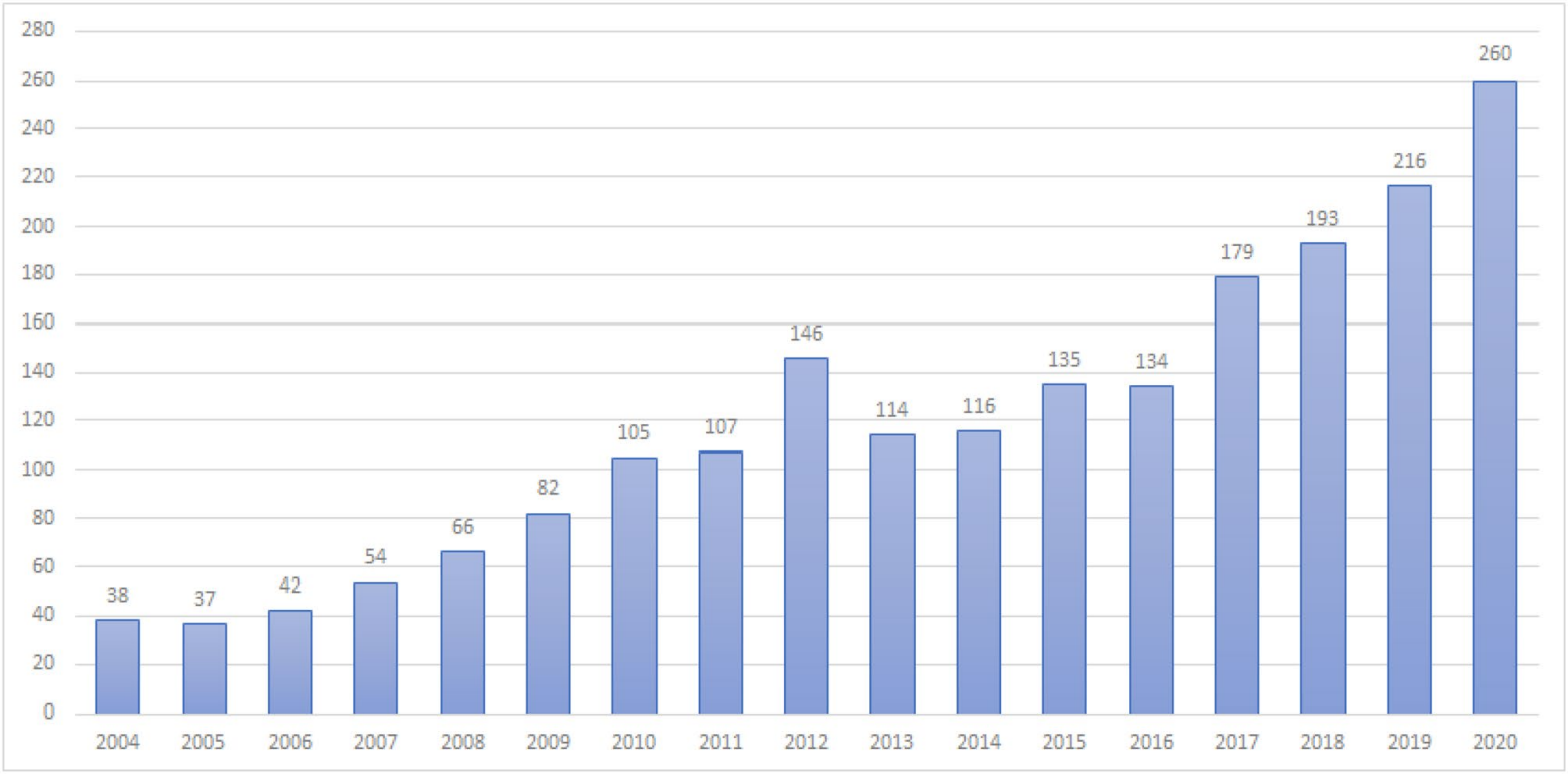
Literature related to vocational education and training from 2004 to 2020.

Indeed, 644 data sources were retrieved from SCI-E, 697 from SSCI, and 693 from CPCI-S. A total of 118 duplicate articles were identified. The cleansing of a sample of data sources was carried out manually by carefully studying the content of abstracts and excerpts from the sources or full versions if they were available. Finally, a total of 2,024 bibliographic citations were obtained (Fig. 1).

## Research tools

Information visualization is the process of representing and visualizing abstract data with the help of computer software, which can enhance researchers' perception of abstract information^31^. Based on the existing literature, visualization analysis can adopt the method of dynamic graphic visualization to reveal the trends, hotspots and frontiers of scientific research. Therefore, information visualization can facilitate researchers to understand and predict the frontiers and trends of scientific research opportunely and break new grounds for new ideas amid complex information.

In this study, the analysis software CiteSpace 5.7 was used to conduct research cooperation, cited references and co-occurrence analysis of keywords in literature pertinent to vocational education and training. CiteSpace is an important software in bibliometrics^32^. CiteSpace visualized knowledge maps can be used to identify, display and predict research trends and elucidate knowledge structure and development^33^. Therefore, CiteSpace's visualized knowledge map was adopted in this study to analyze literature in the field of vocational education and training. The analysis elements included Author, Institution, Country, Cited authors, Cited journals, Cited journals, and Cited references.

A network of cited references, co-authors and keywords co-occurrence can represent the scientific knowledge domains^34^. The network provides a systematic and scientific description of the evolving field of scientific knowledge through knowledge mapping, a novel method of literature analysis, enabling researchers to better understand knowledge structures, research collaborations and the hotspots and trends of research^35^.

## Research process

In this study, a visual analysis of the bibliography of vocational education and training was conducted through CiteSpace. The research process was as follows: Firstly, the basic knowledge cluster of vocational education and training was constructed according to the reference literature of the field. This cluster is necessary for the next steps to identify the main clusters in the knowledge graph and highlight the most influential literature in this field of knowledge. Also, the basic knowledge cluster will help to study the evolution of each cluster, and future trends and identify key literature from a timeline perspective.

Secondly, the hotspots and frontiers of vocational education and training can be identified based on the frequency of the keywords in the related literature. Meanwhile, keyword bursts can also reveal the evolution of vocational education and training and determine the latest research trends. Burst refers to the significant change in the value of a variable over a relatively short period, which is adopted by Citespace to identify research frontiers.

Finally, the researchers, research institutions and countries were visualized to identify the major contributors to the evolution of knowledge in vocational education and training.

## Research limitations

The results obtained may be limited to searching only the Web of Science Core Collection (WOSCC) database, without considering Scopus or other relevant data sources. Also, the sample may not contain sources that directly relate to the topic under study but did not use the corresponding keywords in the article description or other identifiers, and this happens.

## Results

### Knowledge clustering of vocational education and training research

The emergence and development of any new knowledge are based on existing research and findings, and so are vocational education and training. In general, the frontiers of research in a particular discipline can be represented by journal papers to a certain degree, and the cited references form the knowledge base for the journal paper. The important references can be clustered and the co-cited clustering can be determined with the help of specialized computer software, an important step in figuring out the knowledge base for vocational education and training.

The distribution of selected bibliographic citations by year is presented in Fig. 1 and Table 1. First, one should evaluate the relatively uniform growth in the number of works devoted to the topic under study throughout the entire period under study. The only exception is the sharp increase in the number of studies in 2012.

**Table 1:** Settings of the parameters in CiteSpace.

Parameter	Setting
Time slice	2004–2020
Terminology source	Title, abstract, author, plus
Node type	Reference
Strength	Cosine*
Cut	Pathfinder/Pruning the sliced network
Selection	Select the top 50 cited bibliographies in each section

*The formula is calculated as Eq. 1.

In Eq. 1${c}_{ij}$ represents the number of co-occurrences of i and j,${s}_{i}$ is the frequency of occurrence of i, ${s}_{j}$ is the frequency of occurrence of j.

1 $$C{\text{ousine}}\left({c}_{ij},{s}_{i},{s}_{j}\right)= \frac{{c}_{ij}}{\sqrt{{s}_{i}{s}_{j}}}$$

After running CiteSpace, the knowledge mapping was obtained.

Cluster names related to the field of vocational education and training were extracted with the application of MI (Mutual Information). The formula is calculated as Eq. 2, where ${g}_{st}$ is the number of shortest paths from node s to node t; ${n}_{st}^{i}$ is the number of shortest paths through node i among the ${g}_{st}$ shortest paths from node s to node t. 166 clusters were generated based on the co-primer clustering information with 10 main clusters.

2 $$ MI\left( {w,c} \right) = \mathop \sum \limits_{i = 1}^{m} P\left( {c_{i} } \right)\log_{2 } \frac{{P\left( {w |c_{i} } \right)}}{{P\left( w \right)P\left( {c_{i} } \right)}} $$

**Figure 2:**
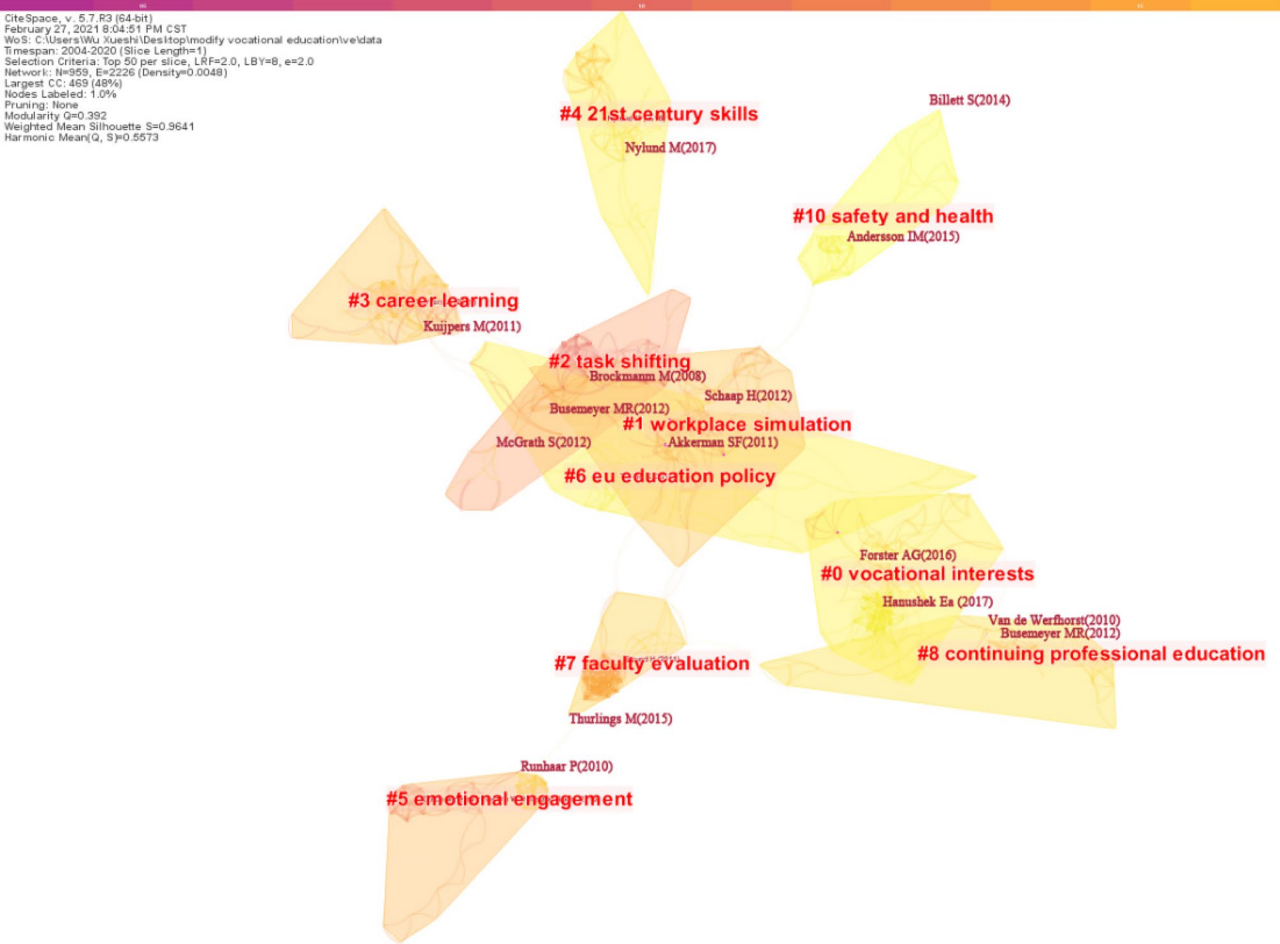
Co-cited literature clusters.

Modularity Q is a measure of visual networks ranging from 0 to 1. The formula is calculated as Eq. 3; $P(w|{c}_{i})$ is the co-occurrence probability of w and c, $P(w)P({c}_{i})$ is the frequency of occurrence of w, $P({c}_{i})$ is the frequency of occurrence of i-type values). The higher the value, the better the network clustering. In general, Modularity Q ranging from 0.3 to 0.8 indicates that network clustering is acceptable. Weighted Mean Silhouette S is a homogeneous indicator of network clustering ranging from − 1 to 1. The larger the Weighted Mean Silhouette S, the higher the clustering homogeneity. In general, Weighted Mean Silhouette S below 0.5 means that the clustering results are acceptable, and above 0.7 means that the clustering results are more reliable^36^. Figure 2 shows the Modularity Q value of 0.392 and the Weighted Mean Silhouette S value of 0.9641 for the visual network in the field of vocational education and training. Weighted Mean Silhouette S values of all 10 major clusters are above 0.8. The above data demonstrate that knowledge mapping is a high-quality clustering of the knowledge domain of vocational education and training.

3 $$Q= \frac{1}{2m}\sum_{ij}({a}_{ij}- {p}_{ij})\sigma ({C}_{i},{C}_{j})$$

Table 2 further provides a more detailed description of each of the knowledge clusters depicted.

**Table 2:** Details of knowledge clusters.

ID	Size	Silhouette	Mean (Year)	Label (MI)
0	62	0.966	2014	Vocational interests (0.75); job quality (0.75); linkage (0.75); occupations (0.75); completion (0.75); routine-biased technological change (0.75); labour market entry (0.75); dual training system (0.75)
1	55	0.879	2010	Workplace simulation (0.64); learning environment (0.64); scaffolding (0.64); workplace simulations (0.64); assessment quality (0.64); webs of reasons (0.64); curriculum model (0.64); socio-cultural theory (0.64); contextualizing vocational knowledge (0.64);
2	42	0.967	2007	Task shifting (0.48); quality perceptions (0.48); conjoint analysis (0.48); assessment (0.48); aspirations (0.48); pharmacy (0.48); skill (0.48); life sciences (0.48); continuing learning pathways (0.48); continuing teacher development (0.48);
3	38	0.997	2010	Career learning (0.07); transformational leadership (0.07); student success (0.07); qualitative methods (0.07); collective learning (0.07); matching (0.07); careers education (0.07);
4	34	0.991	2014	Twenty-first century skills (0.23); social stratification (0.23); general subjects (0.23); citizenship education (0.23); social class (0.23); life skills (0.23);
5	32	0.997	2009	Emotional engagement (0.09); at-risk students (0.09); information acquisition (0.09); social capital (0.09); teacher competences (0.09); latent profile analysis (0.09); latent transition model (0.09);
6	31	0.942	2014	EU education policy (0.32); employers (0.32); skill formation (0.32); labour market transition (0.32); historical institutionalism (0.32); role of the state (0.32); social dialogue (0.32); school-based vocational training (0.32);
7	27	0.936	2010	Faculty evaluation (0.09); variation (0.09); faculty development (0.09); pedagogical content knowledge (0.09); work-related learning (0.09); instructor learning (0.09); vocational areas (0.09);
8	27	0.957	2012	Continuing professional education (0.09); inveduc survey (0.09); vet teachers (0.09); students with disabilities (0.09); labor (0.09); welfare state research (0.09); education systems (0.09); policy preferences (0.09); education policy preferences (0.09); industry currency (0.09);
10	26	1	2014	Safety and health (0.12); technical college (0.12); small business (0.12); workplace safety and health (0.12); occupation (0.12); occupational safety and health (0.12); machine manufacturing (0.12);

It can be concluded from Table 2 that Vocational Interests (#0) ranked first in the knowledge cluster that includes job quality, linkage, occupation, completion, ring-biased Technological change, Labour market entry and dual training system, including 62 literature, most of which were published around 2014. The Weighted Mean Silhouette S value of the cluster is 0.966, indicating the high homogeneity of the 62 literature in the cluster. Among them, General Education, Vocational Education, and Labor-Market Outcomes over the Life-Circle, by Hanushek et al.^37^ on *Journal of Human Resources*, are the articles with the highest citation (60% of the articles in this cluster cited this article); Vocational Education and Employment over the Life Cycle by Forster et al.^38^ on *Sociological Science*, ranks the second (34% of the studies in the cluster cited this article); Educational Systems and the Trade-Off between Labor Market Allocation and Equality of Educational Opportunity by Bol and Van de Werfhorst^18^ on *Comparative Education Review* ranks the third (27% of the studies in the cluster cited this article).

The second clustering is Workplace Simulation (#1) with 55 articles, and the Weighted Mean Silhouette S value of the cluster is 0.879. The most cited article was Students' Learning Processes during School-based Learning and Workplace Learning in Vocational Education: A Review by Schaap et al.^40^ on *Vocations and Learning*. Forty percent of the studies in the cluster cited this article. The third cluster, Task Shifting (#2), consisted of 42 articles. The Weighted Mean Silhouette S value of the cluster is 0.967, a high homogeneity. The detailed information of each major cluster is shown in Table 3.

**Table 3:** The most active citation cluster.

Coverage	Author (Year)	Journal	Articles
Clusters #0	Vocational interests		
60%	Hanushek et al.^37^	J Hum Resour	General Education, Vocational Education, and Labor-Market Outcomes over the Life-Circle
34%	Forster et al.^38^	Sociol Sci	Vocational Education and Employment over the Life Cycle
27%	Bol and Van de Werfhorst^18^	Comp Educ Rev	Educational Systems and the Trade-Off between Labor Market Allocation and Equality of Educational Opportunity
23%	Protsch and Solga^19^	J Educ Work	The social stratification of the German VET system
19%	Forster and Bol^30^	Soc Sci Res	Vocational education and employment over the life course using a new measure of occupational specificity
18%	Malamud and Pop-Eleches^39^	Rev Econ Stat	General education versus vocational training: evidence from an economy in transition
Clusters #1	Workplace simulation		
40%	Schaap et al.^40^	Vocat Learn	Students’ Learning Processes during School-Based Learning and Workplace Learning in Vocational Education: A Review
25%	Akkerman and Bakker^41,42^	Rev Educ Res	Boundary Crossing and Boundary Objects
24%	Tynjälä^28,29^	Educ Res Rev-Neth	Perspectives into learning at the workplace
24%	De Bruijn and Leeman^25^	Teach Teach Educ	Authentic and self-directed learning in vocational education: Challenges to vocational educators
18%	Baartman and De Bruijn^43^	Educ Res Rev-Neth	Integrating knowledge, skills and attitudes: Conceptualizing learning processes towards vocational competence
Clusters #2	Task shifting		
36%	McGrath^44^	Int J Educ Dev	Vocational education and training for development: A policy in need of a theory?
33%	Brockmann et al.^45^	Vocat Learn	Competence-Based Vocational Education and Training (VET): the Cases of England and France in a European Perspective
29%	Biemans et al.^46^	J Vocat Educ Train	Towards competence‐based VET: dealing with the pitfalls
26%	Biemans et al.^47^	J Vocational Ed Trai	Competence-based VET in the Netherlands: background and pitfalls
Clusters #3	Career-learning		
29%	Kuijpers et al.^48^	J Vocat Behav	The relationship between learning environment and career competencies of students in vocational education
18%	Winters et al.^26^	J Vocat Educ Train	What are vocational training conversations about? Analysis of vocational training conversations in Dutch vocational education from a career learning perspective
Clusters #4	21th century skills		
24%	Nylund and Rosvall^27^	J Curriculum Stud	A curriculum tailored for workers? Knowledge organization and possible transitions in Swedish VET
24%	Nylund et al.^22^	J Educ Policy	The vocational–academic divide in neoliberal upper secondary curricula: the Swedish case
18%	Ledman et al.^20^	J Vocat Educ Train	Gendered distribution of ‘knowledge required for empowerment’ in Swedish vocational education curricula?
18%	Lappalainen et al.^21^	Gender Educ	Gendered divisions on classed routes to vocational education

In addition, widely recognized studies can be identified based on the number of citations. The top 3 studies with the highest citations (over 20 citations) are as follows: General Education, Vocational Education, and Labor-Market Outcomes over the Life-Cycle published by Hanushek et al.^37^ on *Journal of Human Resources* (37 citations); Students' Learning Processes during School-based Learning and Workplace Learning in Vocational Education: A Review by Schaap et al.^40^ (22 citations) on *Vocations and Learning*; Vocational Education and Employment over the Life Cycle by Forster et al.^38^ on *Sociological Science* (20 citations).

Given the lack of expansion of the thematic field of research after 2013, as shown further in tables, it can be assumed that the Vocational Interests knowledge cluster has focused the most interest of researchers and perhaps provided the most topics for further in-depth research. It is the state of the labor market and the relationship with employment that has received the most attention. researchers in connection with vocational education.

### Distribution of countries

**Figure 3:**
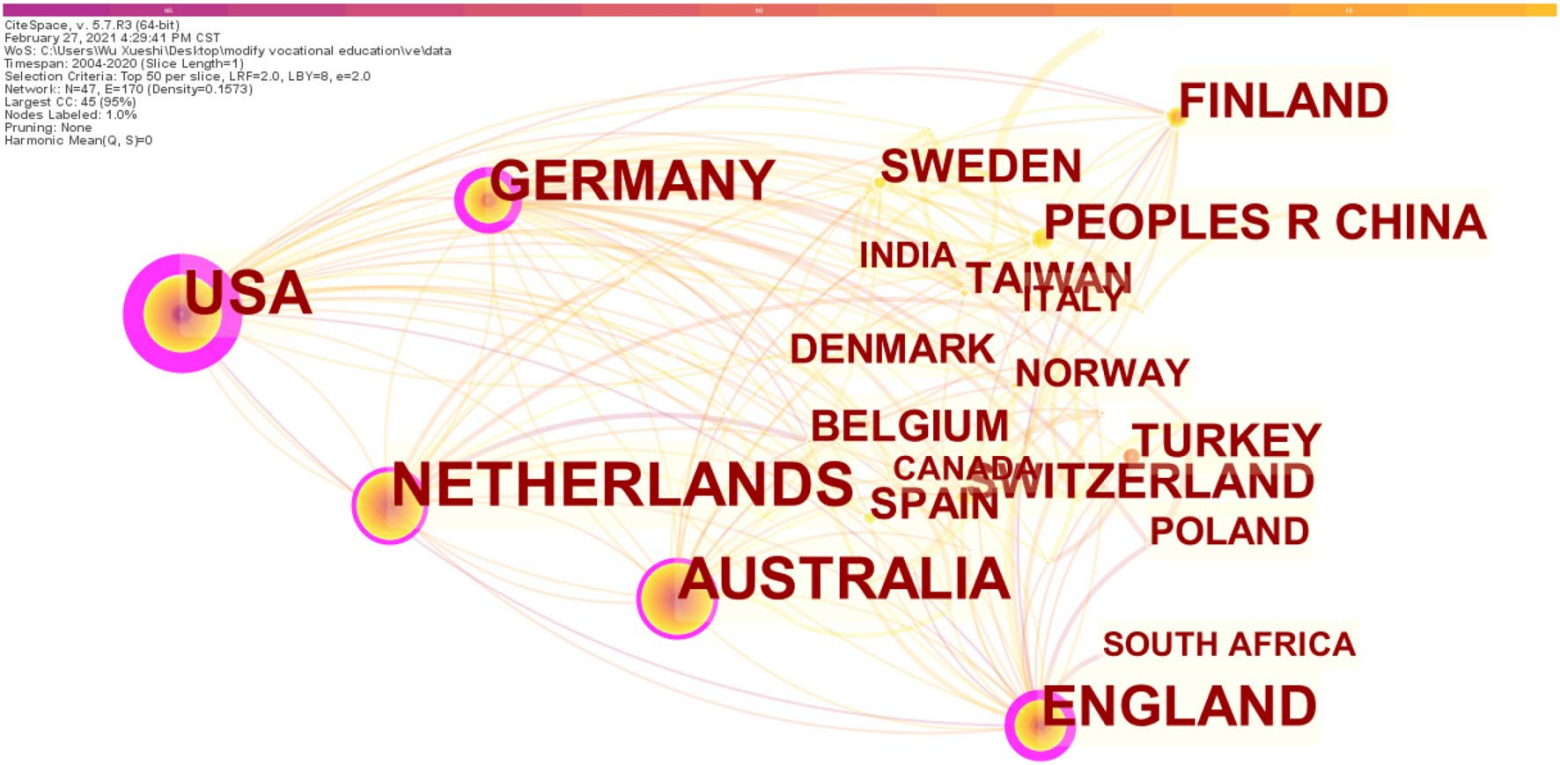
Network of countries distribution for VET.

The number of papers published by different countries and their academic influence can be elaborated on in Table 4. It can be inferred that the United States was the most productive among the top 10 countries followed by the Netherlands and Australia in the field of vocational education and training with 260 papers published from 2004 to 2020, accounting for about 14% of all literature. However, China ranked sixth with 88 papers, accounting for 4.8% of all papers, far lower than that of the United States. In terms of betweenness centrality value, the USA (0.67), England (0.36) and Germany (0.27) ranked in the top three, indicating a significant academic influence on the field of vocational education and training. Betweenness centrality indicates the strength of a node's influence on the flow of information in the graph. This is a measure of the influence of a separate node in a whole network^33^. The United States ranked first regarding the academic influence in the field of vocational education and training research while China is still in a relatively disadvantaged position. A more complete visual representation of the distribution of academic influence by country is presented in Fig. 3.

**Table 4:** VET research distribution by countries.

Country/region	Frequency
USA	260
Netherlands	251
Australia	217
England	159
Germany	154
Peoples R China	88
Finland	86
Switzerland	76
Turkey	60
Sweden	55

Country	Centrality
USA	0.67
England	0.36
Germany	0.27
Australia	0.16
Netherlands	0.13
South Korea	0.1
Russia	0.09
Switzerland	0.05
Peoples R China	0.04
Italy	0.04

Country	Burstiness
Turkey	12.38
England	10.56
Italy	7.09
Australia	6.12
China	5.88
Netherlands	5.87
Finland	5.21
South Korea	5.08
Switzerland	4.01
Sweden	3.98
New Zealand	3.72

Burstiness (Table 4 the last section) is an increase and decrease in activity or frequency of publications that disrupts the continuity or pattern of distribution. In this case, the higher this indicator, the more uneven the participation of publications from this university in the research field being studied. The Burstiness results of countries more active in the field of vocational education and training (Table 4, Burstiness section) showed that: Turkey ranked first with a value of 12.38, followed by England which has been more active in this field. However, although Sweden and New Zealand are not very active in this field, academic attention has been drawn to them.

### Distribution of research institutions

**Figure 4:**
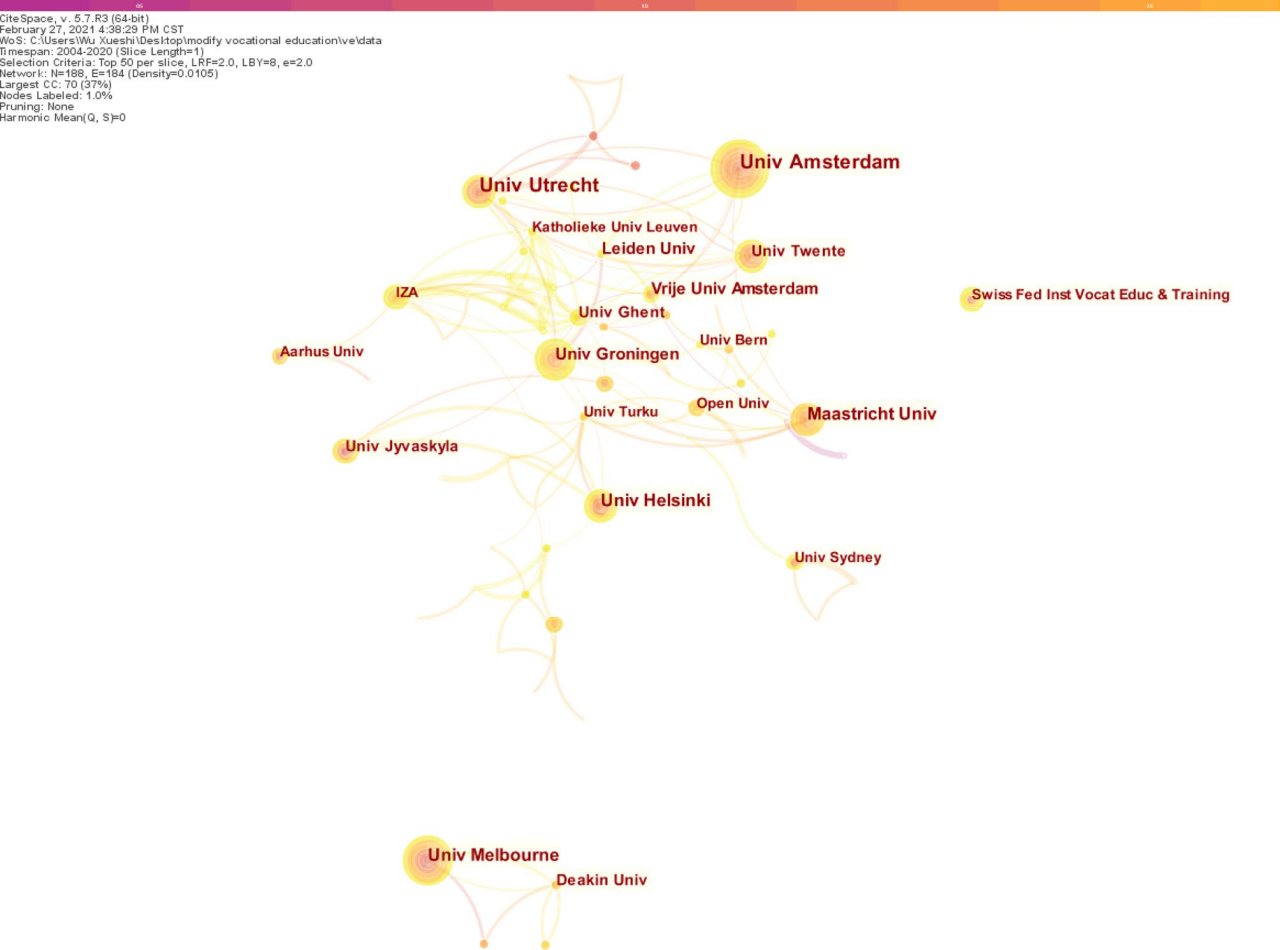
Network of institutions for VET research.

In terms of the production of research institutions in the field of vocational education and training, Univ Amsterdam ranked first with 30 articles, followed by Univ Utrecht (28 articles) and Univ Melbourne (22 articles) (Fig. 4).

However, the ranking of research institutions based on betweenness centrality demonstrated significant influence from other research centers. The top three universities with betweenness centrality were IZA (0.09), Univ Turku (0.09) and Univ Helsinki (0.07), indicating the importance and influence of these three universities in the field of vocational education and training. Regarding the post surge capacity, Gazi Univ was in the lead with a surge of 5.52, followed by Leiden Univ (5.02) and Univ Utrecht (4.24). See Table 5 for details.

**Table 5:** Contributing institutions by frequency, centrality and burst.

Institutions	Frequency
Univ Amsterdam	30
Univ Utrecht	28
Univ Melbourne	22
Maastricht Univ	22
Univ Helsinki	22
Univ Groningen	18
Leiden Univ	16
Vrije Univ Amsterdam	15
Deakin Univ	14
Univ Ghent	14

Institutions	Centrality
IZA	0.09
Univ Turku	0.09
Univ Helsinki	0.07
Univ Groningen	0.06
Univ Utrecht	0.04
Harvard Univ	0.04
Univ Amsterdam	0.03
Maastricht Univ	0.03
Open Univ	0.03
Natl Res Univ Higher Sch Econ	0.03

Institutions	Burstiness
Gazi Univ	5.52
Leiden Univ	5.02
Univ Utrecht	4.24
Univ Twente	3.8
Eindhoven Univ Technol	3.44
Marmara Univ	3.35
Kings Coll London	3.19
Univ Oxford	3.18
Univ Melbourne	3.12
Hacettepe Univ	3.01

### Cited journals

**Figure 5:**
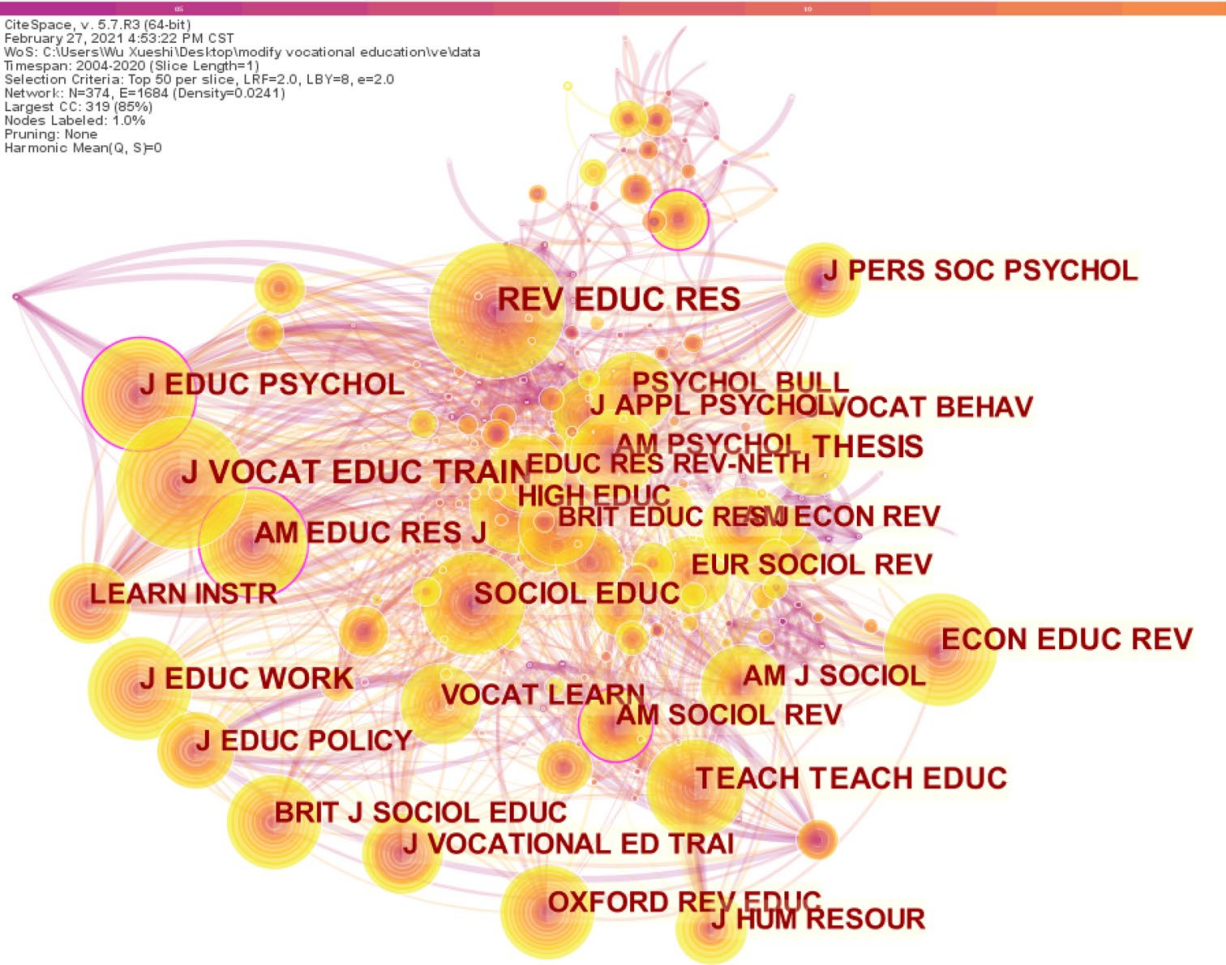
Cited journals network.

In the citation network of journals, the larger the circle, the higher the citation frequency (Fig. 5).

Totally 233 pieces of literature on *J Vocat Educ Train* were cited; 208 on *Rev Educ Res*; 181 on *Thesis Elev*; and 156 on *Econ Educ Rev* (Table 6).

**Table 6:** Cited journals by frequency, centrality and burst.

Source	Frequency
J Vocat Educ Train	233
Rev Educ Res	208
Thesis Elev	181
Econ Educ Rev	156
J Educ Work	150
Teach Teach Educ	149
J Educ Psychol	144
Sociol Educ	139
Am Educ Res J	136
Brit J Sociol Educ	126
Vocat Learn	122
J Vocat Behav	121

Source	Centrality
J Educ Psychol	0.15
Am Educ Res J	0.14
Am Sociol Rev	0.13
Rev Educ Res	0.09
Am J Sociol	0.09
Am Econ Rev	0.09
J Vocat Educ Train	0.08
Econ Educ Rev	0.08
Sociol Educ	0.08
Teach Teach Educ	0.07
Learn Instr	0.07

Source	Strength
Thesis elev	31.74
Soc Sci Res	12.51
Comp Educ	12.27
Teach Teach	11.49
Econ J	11.25
J Labor Econ	10.63
Res Soc Strat Mobil	10.04
Educ Researcher	9.56
J Ed Work	9.11
Rev Econ Stat	9

However, from 2004 to 2020, *Thesis Elev* ranked first in burst detection with a burst value of 31.74. Other journals with relatively high emergent detection values include SOC SCI Res, Comp Educ, Teach Teach, Econ J, J Labor Econ, etc. These journals mainly come from the fields of psychology, sociology, economics and pedagogy, the source of knowledge in the field of vocational education and training.

### Hotspots and trends of the research on vocational education and training

The research hotspot is the focus of researchers' attention shared by a group of interrelated papers in a relatively short period. Keywords are the gist and soul of an academic paper, a highly summarized and refined research problem, and an important index of research hotspots. Therefore, the research hotspots and main characteristics of a certain field can be abstracted from the change in keyword frequency. In this study, "Keyword" was selected from the CiteSpace node types for Keyword co-occurrence network analysis. The larger the node, the more important the node.

In terms of keywords frequency (Table 6), related research mainly focused on vocational education and training in vocational education, the transition, inequality, gender, perception, attitude, and the program, work, school, and skill, among which, the keyword "vocational education" ranked first for appearing 399 times, followed by education (234 times) and vocational education and training (181 times). Betweenness centrality higher topics include health, adolescent, perspective, gender, employment, model, etc.

**Figure 6:**
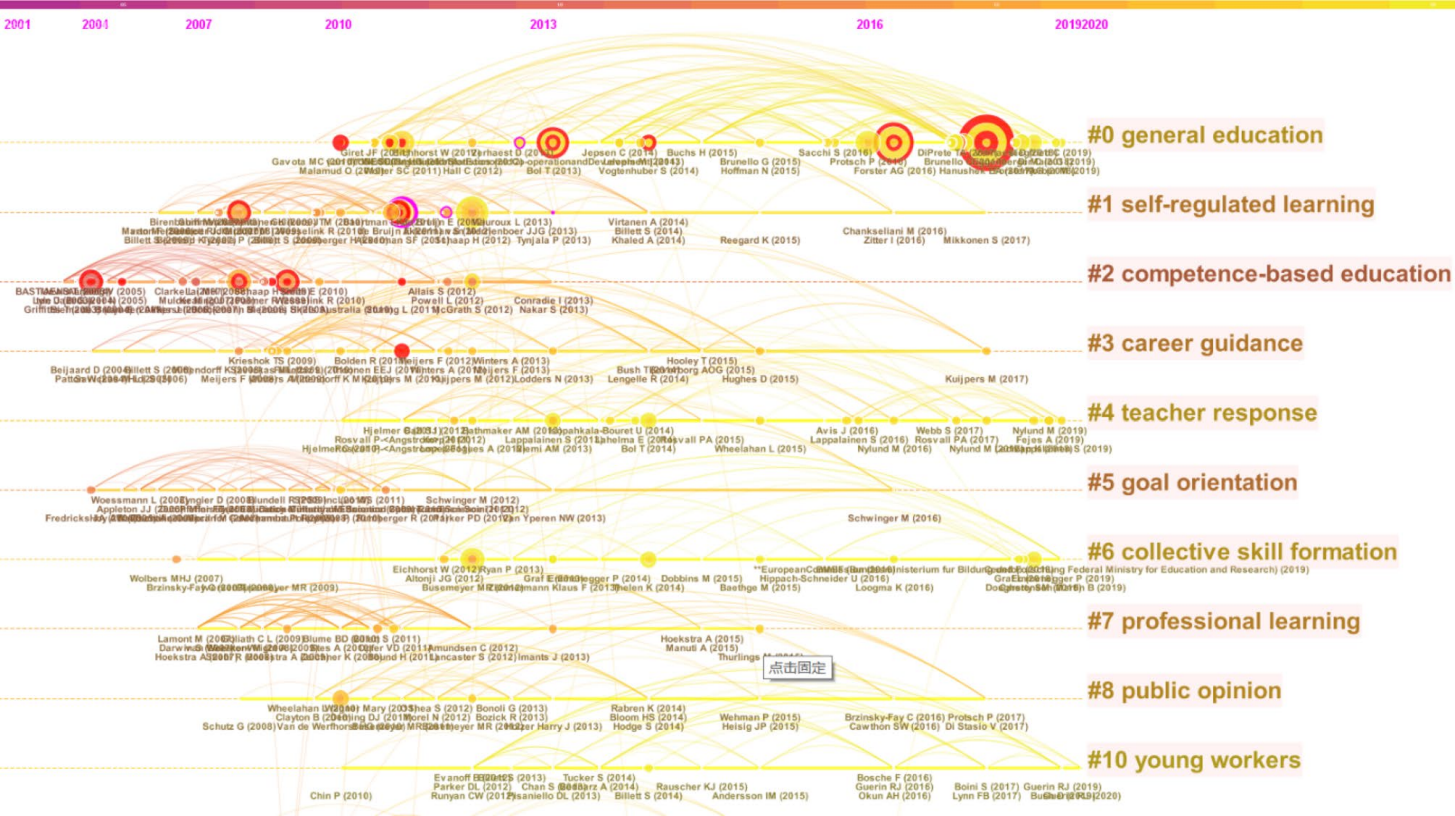
Timeline of co-citation clusters from 2004 to 2020.

Meanwhile, keyword selection was carried out to clearly show the research hotspots in different years and their interrelation and evolution. Since none of the keywords identified during the study were localized for the period after 2013, we can conclude that the thematic field of research after this time developed almost exclusively intensively, and not extensively, that is, the research hotspots that had already been emphasized earlier were explored (Fig. 6).

Keywords that appeared more than 25 times were selected and checked for betweenness centrality, as shown in Table 7.

**Table 7:** Main research topics by year.

Year	Keyword	Frequency	Centrality
2004	Vocational education	399	0.06
2004	Transition	92	0.05
2004	Inequality	54	0.05
2004	**Gender**	**50**	**0.1**
2004	Perception	40	0.01
2004	Attitude	35	0.03
2004	Program	30	0.02
2005	Work	107	0.05
2005	School	101	0.04
2005	Skill	86	0.06
2005	Knowledge	80	0.02
2005	**Employment**	**76**	**0.1**
2005	Performance	71	0.05
2005	Impact	66	0.03
2005	**Health**	**61**	**0.13**
2005	Youth	45	0.03
2005	Children	36	0.01
2005	Achievement	34	0.02
2006	Education	234	0.06
2006	**Adolescent**	**72**	**0.13**
2006	Motivation	52	0.07
2006	System	39	0.06
2006	Return	33	0.02
2006	Labor market	31	0.01
2006	Competence	31	0.06
2006	Vocational education	29	0.01
2006	Strategy	25	0.04
2007	Vocational education and training	118	0.04
2007	**Model**	**65**	**0.1**
2007	Technical education	46	0.03
2007	Behavior	38	0.02
2008	Student	105	0.04
2009	Higher education	79	0.04
2009	Policy	55	0.05
2009	Perspective	38	0.13
2009	Curriculum	37	0.04
2010	Teacher	55	0.01
2010	Apprenticeship	30	0.01
2012	Participation	27	0.01
2013	High school	25	0.01

Significant values are in bold.

## Discussion

Compared with previous research^23,24^, this research uses CiteSpace V to analyze the research hotspots and research frontiers of vocational education and training from 2004 to 2020, and finds that:

First, the annual volume of research literature is steadily increasing, but the growth rate is relatively low. This is the same as the result of Hui's research^23^. The reasons for this result are as follows: The first is that the academic level and subject status of vocational and technical education are not yet mature, and its knowledge fields and subject boundaries are not clear enough, which causes the subject of vocational and technical education to face multiple identity crises^27,28^. The second is that the interdisciplinary nature of vocational education makes its research power scattered in many disciplines such as pedagogy, economics, management, and sociology, while there are fewer academic groups specializing in vocational and technical education^3,7^.

Second, from the perspectives of research countries, institutions, authors and journals, the main drivers of research in the field of vocational education and training come from the United States, the Netherlands and Australia, with Univ Amsterdam, Univ Utrecht and Univ Melbourne as the leading institutions. De Bruijn from Utrecht University, Christopher Winch from University of Westminster Univ Westminster, Pietty Runhaar from Deakin University, Martin Mulder from King's College, and Derek G Shendell from Rutgers State University, were the major contributors to vocational education and training. Literature on J VOCAT Educ Train was the most highly cited (233 times), followed by the top five journals including Rev Educ Res (208 times), *Thesis Elev* (181 times), Econ Educ Rev (156 times) and J Educ Work (150 times). This is different from Yu and Zhou's research results^24^. Through analysis of 719 literature titles, Li proposed that the main research countries for vocational education and training are European countries and the United States^12^. The reasons for the difference between the two may be: The first is the sample size. This study uses 3844 literature titles in the Web of Science database, which has a larger sample size coverage and more effective results; while Li's research has only 719 literature titles and a smaller sample range. The second is the time frame. This research uses 15 years of literature from 2004 to 2020, which represents the latest research characteristics in the field of vocational education and training; while Li uses literature from 2000 to 2009, which can only represent the characteristics of previous research.

Third, in terms of the most popular research topics, growth, vocational education and training, politics, university, secondary education, the environment, China, and other aspects of inequality took the lead from 2004 to 2015, and after 2015^20,24^. Other researchers agree with the results obtained in that study showing that the field started to focus on inequality, the teacher, professional development, engagement, program, self-efficacy, high school, the predictor and labor market, among which, the fields of engagement, program, self-efficacy, high school, predictor and labor market are still active and may become future research directions^16,24^. This is consistent with Hui's research results^23^. Technological changes and socio-economic development require vocational education and training to gradually shift the focus to students’ cross-industry abilities, and to pay close attention to the dynamic needs of the labor market. In addition, this has a certain relationship with the gradual change of vocational education research from macro to meso and micro.

Although an effective visual analysis of the relevant studies in the field of international vocational education and training from 2004 to 2020 was conducted, the obtained data cannot fully represent the overall picture of the development of international vocational education and training. Limited by research conditions, the related studies of international vocational education and training from the Web of Science were downloaded from 2004 to 2020. Significant potential for future research is to explain the observed spillovers in the influence and contributions of different countries and institutions over significant periods and how they change due to market influences, changes in technology, and other possible factors. Future researchers are encouraged to use a wider range of journals over a longer period.

## Conclusions

By drawing the scientific knowledge map of international Vocational Education and Training from 2004 to 2020, this paper intuitively demonstrates the growth law of papers, knowledge sources, author contributions, institutional cooperation and national cooperation in this research field. It also analyzes the research hotspots in the field of vocational education and training, and draws the following conclusions from a comprehensive perspective:

- Paper growth law. From 2004 to 2009, the development of Vocational Education and Training research was relatively slow. Since 2010, new Vocational Education and Training research has shown a vigorous development trend. The amount of new media research will reach its peak in 2020. The author predicts that in the future, Vocational Education and Training research will continue to show a trend of vigorous development.
- Knowledge source. In the field of Vocational Education and Training, 12 journals have been cited more than 120 times. These journals mainly focus on psychology, sociology, economics and pedagogy. This shows that the knowledge in the field of Vocational Education and Training mainly comes from the above four disciplines.
- Author contribution. Hanushek Ea, Forster AG, Bol T, Schaap, Akkerman, McGrath, Brockmann, Kuijpers, Nylund and other highly cited authors have provided high-quality papers and belong to high-impact authors.
- Institutional cooperation. The most researched institution in the field of Vocational Education and Training is Univ Amsterdam (30 articles), followed by Univ Utrecht (28) and Univ Melbourne (22 articles), Maastricht Univ (22 articles), and Univ Helsinki (22 articles). On the whole, there is a lack of cooperation and exchanges between institutions, and no large-scale cooperation network has been formed.
- Country cooperation. The country with the most research in the field of Vocational Education and Training is the USA (260 articles), followed by NETHERLANDS (251) and AUSTRALIA (217). Although there are many research results in the field of Vocational Education and Training in various countries, the cooperation network between countries needs to be strengthened urgently.
- Research hotspots. The relatively high intermediary centrality in the field of Vocational Education and Training is health (0.13), adolescent (0.13), gender (0.1), employment (0.1), and model (0.1). This shows that the above content is a research hotspot in this field.

## Data Availability

Data will be available from the corresponding author (Xueshi Wu) on request.
